# GARS: Genetic Algorithm for the identification of a Robust Subset of features in high-dimensional datasets

**DOI:** 10.1186/s12859-020-3400-6

**Published:** 2020-02-11

**Authors:** Mattia Chiesa, Giada Maioli, Gualtiero I. Colombo, Luca Piacentini

**Affiliations:** 1 Unit of Immunology and Functional Genomics, Centro Cardiologico Monzino IRCCS, Via Carlo Parea, 4, 20138 Milan, Italy; 2 Department of Electrical, Computer and Biomedical Engineering, University of Pavia, Pavia, Italy

**Keywords:** Genetic algorithms, Feature selection, Machine learning, Omics, High-dimensional data

## Abstract

**Background:**

Feature selection is a crucial step in machine learning analysis. Currently, many feature selection approaches do not ensure satisfying results, in terms of accuracy and computational time, when the amount of data is huge, such as in ‘Omics’ datasets.

**Results:**

Here, we propose an innovative implementation of a genetic algorithm, called GARS, for fast and accurate identification of informative features in multi-class and high-dimensional datasets. In all simulations, GARS outperformed two standard filter-based and two ‘wrapper’ and one embedded’ selection methods, showing high classification accuracies in a reasonable computational time.

**Conclusions:**

GARS proved to be a suitable tool for performing feature selection on high-dimensional data. Therefore, GARS could be adopted when standard feature selection approaches do not provide satisfactory results or when there is a huge amount of data to be analyzed.

## Background

In machine learning, the feature selection (FS) step seeks to pinpoint the most informative variables from data to build robust classification models. This becomes crucial in the Omics data era, as the combination of high-dimensional data with information from various sources (clinical and environmental) enables researchers to study complex diseases such as cancer or cardiovascular disease in depth [1–4]. Given the amount and sophistication of data, accurate prediction, for example, of the nature of the disease and/or the outcome of patients is difficult, but the design of high-performance classification models through the application of machine learning is strongly required.

There are several methods available for performing FS, which are generally grouped into three main categories: (i) filter-based methods that rely on univariate statistics, correlation or entropy-based measurements; (ii) wrapper methods, which combine the search algorithms and classification models; and (iii) *embedded* methods, where the FS is realized during the construction of the classifier. Even though they are often fast and easy-to-use on low to medium size data, these techniques have however substantial disadvantages: the filter-based methods ignore the relationship between features, whereas the wrapper methods are prone to over-fitting and get stuck in local optima [5]. Furthermore, wrapper and, to a lesser extent, embedded methods present a high computational complexity, increasing serious constraints when dealing with a high number of features (> 15,000), i.e. in Omics datasets; this makes necessary to precede these methods with a previous filter-based method or standard pre-processing, in order to be effective [6, 7]. Another way of categorizing FS methods is to consider their algorithmic aspect, specifically as a search problem, thus classifying FS as exhaustive, heuristic and hybrid search methods [8]. Exhaustive search is very limited in practice because these methods try all possible feature combinations of the total original features, thus, making computational calculations too heavy to be effectively accomplished. Conversely, heuristic search aims to optimize a problem by improving iteratively the solution based on a given heuristic function, whereas hybrid methods are a sequential combination of different FS approaches, for example those based on filter and wrapper methods [9].

A specific class of wrapper methods is represented by optimization approaches, inspired by natural selection, such as population-based or Genetic Algorithms (GAs) [10]. GAs are adaptive heuristic search algorithms that aim to find the optimal solution for solving complex problems. Briefly, a GA tries and assesses the goodness of a set of candidate solutions, called chromosomes, simulating the Darwinian law of the “survival of the fittest”. Chromosomes are a string of a set of variables. Specifically, a GA is composed of five steps: (1) generation of a random set of chromosomes (ˈPopulationˈ); (2) evaluation of each chromosome by a score that reflects how good the solution is (ˈFitness Functionˈ); (3) ‘Selection’ of chromosomes with the highest fitness score; (4) ˈCrossover’ between pairs of chromosomes at points chosen from within the chromosomes to generate offspring (‘Reproduction’); and (5) ‘Mutation’ with a low random probability. The last three are called “evolutionary” steps. At the end of this process, a new “evolved” chromosome population is obtained. To find the optimal solution this scheme is repeated several times until the population has converged, i.e., new offspring are not significantly different from the previous generation.

These optimization strategies ensure better performance, in terms of classification accuracy, than simpler FS techniques such as filter-based or deterministic wrapper methods. In addition, GAs are capable to search the optimal solution on high-dimensional data composed of mutually dependent and interacting attributes. Nonetheless, GAs are more computationally expensive. Moreover, GAs, like every wrapper method, are more prone to overfitting, because a specific classifier is built to assess both the goodness of the fitness function and classification accuracy [5]. To do this, popular methods are based on Support Vector Machines [11] and Random Forest [12]. For these reasons, GAs have not been widely used for performing FS, despite their high potential.

To overcome these limitations, here, we propose an innovative implementation of such algorithms, called Genetic Algorithm for the identification of a Robust Subset (GARS) of features. GARS may be applied on multi-class and high-dimensional datasets, ensuring high classification accuracy, like other GAs, taking a computational time comparable with basic FS algorithms.

## Results

### GARS implementation

**Fig. 1:**
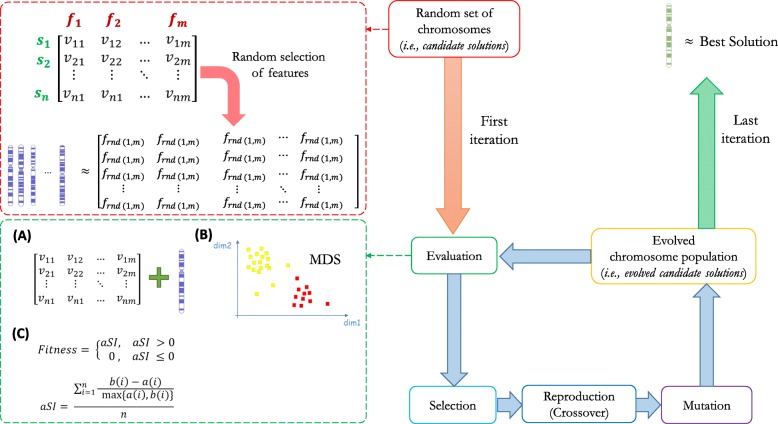
Block diagram of the GARS workflow. The first population of chromosomes (red block) is created by randomly selecting sets of variables (see the red box on the left). Then, each chromosome is assessed (green block). To do this (see green box on the left), we designed a fitness function that (**A**) extracts for each sample the values of the variables corresponding to the chromosome features, (**B**) uses them to perform a Multi-Dimensional Scaling (MDS) of the samples, and (**C**) evaluates the resulting clustering by the average Silhouette Index (aSI). Finally, to obtain a new evolved population, the Selection (light blue block), Reproduction (blue) and Mutation (purple) steps are implemented. This process, iteratively repeated several time, allows to reach the optimal solution. *f* = feature, *s* = sample, *v* = value of a features in a sample, *n* = total number of samples, *m* = total number of features, *rnd (1,m)* = random integer between 1 and m, *i* = specific sample, *a(i)* = average dissimilarity of i with respect to all other samples within the same class, *b(i)* = the lowest averaged distance of i to all samples belonging to any other class, *aSI* = average Silhouette Index, and *MDS* = Multi-Dimensional Scaling

A specific GA is characterized by a custom implementation of the chromosome structure and the corresponding fitness function. Let assume we have a dataset *D* with *n* samples (s_1_, s_2_,..., s_n_) and *m* features (f_1_, f_2_,..., f_m_). In GARS, we define the chromosome as a vector of unique integers, where each element represents the index (1 to *m*) of a specific feature in the dataset. The length *l* of each chromosome, with *l* < *m*, corresponds to the length of the set of features to be selected. A chromosome population is, thus, a matrix *l × k*, where *k* is the number of chromosomes (Fig. 1). The first population must be randomly generated.

A specific and distinctive characteristic of GARS implementation is the way to evaluate the fitness of each chromosome. This is accomplished in two consecutive steps: first, a Multi-Dimensional Scaling (MDS) of the examined samples is performed using the chromosome features. Then, the averaged Silhouette Index (aSI, [13]) is calculated on the sample coordinates (first 2 dimensions) obtained by MDS:

1 $$ aSI=\frac{\sum_{i=1}^n\frac{b(i)-a(i)}{\max \left\{a(i),b(i)\right\}}}{n} $$

where *i* is a sample, *n* is the total number of samples, *a(i)* is the average dissimilarity of *i* with respect to all other samples within the same class, and *b(i)* is the lowest averaged distance of *i* to all samples belonging to any other class. Finally, the negative values of aSI are set to 0 (see the flowchart in Fig. 1):

2 $$ Fitness=\left\{\begin{array}{c} aSI,\kern0.5em aSI>0\\ {}\kern0.75em 0,\kern1.00em aSI\le 0\end{array}\right. $$

In this way, the maximum fitness score is equal to 1 (i.e., the score that can be assigned to a chromosome with the maximum discrimination power), while the minimum fitness score is 0 (i.e., a chromosome with no discrimination power). For fitness score = 1, all samples are correctly allotted to their class and each group of samples is very far from each other. For fitness score = 0, the sample groups cannot be distinguished.

The evolutionary steps implemented in GARS are accomplished by the most frequently used methods and consist of an elitism step, coupled with the *Tournament* or the *Roulette Wheel* selection methods, followed by the *one-point* or *two-points* crossover [14, 15]. In addition, the mutation step is carried out by replacing a specific chromosome element with a random number, not present in that chromosome, in the range 1 to *m*.

### Performance and comparison with other FS methods

To evaluate the performance of GARS, we implemented three machine learning analyses, testing our GA against an univariate filter-based method, called Selection By Filtering (SBF) [5], a wrapper method, consisting of a Recursive Feature Elimination (RFE) strategy [16], an embedded method called LASSO (Least Absolute Shrinkage and Selection Operator) regression [17], and two GAs, where the fitness function was calculated by a Support Vector Machine (svmGA) [18] and a random forest classifier (rfGA) [19], respectively (see Methods).

The first and the second analyses aimed to select features in binary classification problems, using a low-dimensional (henceforth, ‘binary low-dimension’ dataset) and a mid-dimensional dataset (‘binary mid-dimension’), respectively (see Methods). The former dataset was obtained by a miRNA-Seq experiment, investigating the miRNAome dysregulation in cervical cancer tissues [20]; the latter resulted from a Nuclear Magnetic Resonance (NMR) spectrometry experiment, in which hundreds of urinary metabolic features were studied in acute kidney injury [21]. In the last analysis, each method was tested on several multi-class classification problems, using high-dimensional data (‘multi high-dimension’ dataset) downloaded from the Genotype-Tissue Expression portal (GTEx, https://gtexportal.org/home/) [22, 23]. In particular, we used RNA-Seq expression data from 11 brain regions (see Methods).

#### Low-dimensional dataset in a binary classification problem

The ‘binary low-dimension’ dataset allows us to evaluate the performance in an easy binary classification problem, where the number of features is relatively small, and groups are well separated (see Additional file 1: Fig. S1, panel A). This dataset, after pre-processing steps (see Methods), was composed of 58 samples and 168 features. We randomly split the dataset into two subsets: a ‘learning dataset’, composed of 50 samples (25 tumors, T, and 25 non-tumor, NT), and an ‘independent test set’, composed of the remaining 8 samples (4 T and 4 NT). The range of desired chromosome features was set from 5 to 20, for GARS and RFE. As for the three GAs, we chose reasonable and frequently used GA parameters, setting the probability of mutation to 0.1, the crossover rate to 0.8, the number of iteration to 100, the number of chromosomes to 100, and the number of chromosomes kept by elitism to 2. We considered ‘T’ as the positive class.

**Fig. 2:**
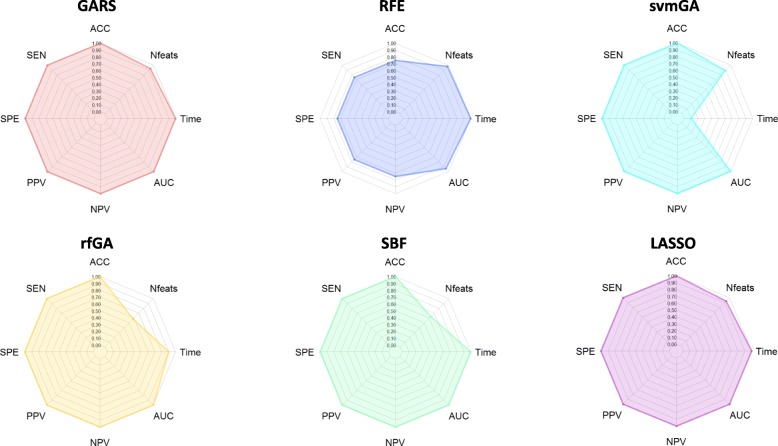
Radar plots that summarize the performance of the different algorithms tested in a ‘binary low-dimension dataset’. To test the efficacy of each algorithm, we calculated ACC = Accuracy, SEN = Sensitivity, SPE = Specificity, PPV = Positive Predictive Value, NPV = Negative Predictive Value, AUC = Area Under ROC Curve, and Nfeats = n. of selected features on the independent test set. To evaluate the efficiency of each algorithm, we measured the average learning time for each cross-validation fold (Time). To get an overall assessment of the algorithm performance, we calculated the area of the polygon obtained connecting each point of the aforementioned measurements: the wider the area, the better the overall performance. GARS (red chart) and LASSO (purple chart) covered 98% of the total area, SBF (green chart) 91%, rfGA (yellow chart) 87%, svmGA (light blue chart) 76% and RFE (blue chart) 70%

Results obtained after the classification analysis are summarized in Table 1. Overall, GARS and LASSO outperformed the other four FS methods, by identifying the smallest feature set (*n* = 14) capable of ensuring the maximum accuracy, sensitivity, specificity, Negative Predicted Value (NPV), Positive Predicted Value (PPV) and Area Under ROC Curve (AUC). Notably, the feature sets selected by GARS and LASSO are 1.5 to 6 times smaller than the subsets identified by svmGA, SBF, and rfGA (Table 1). Compared to GARS, the two out of three fastest methods (i.e., RFE and SBF) did not reach an equally high classification accuracy or else selected far more numerous features, respectively. On the other hand, the other two most accurate algorithms (i.e., rfGA and svmGA) needed several hours to identify a set of features. Only LASSO ensured a very short execution time and a small number of features. To jointly assess the improvement of efficacy and efficiency over the other algorithms, we used radar charts displaying the performance metrics of the ongoing programs (Fig. 2). These highlighted that, due to its short computational learning time (about 4 min per fold), high classification performances, and the small number of resulting features, GARS and LASSO achieved the highest score covering 98% of the total area.

**Table 1:** Performance evaluation, testing FS methods on the ‘binary low-dimension’ dataset

	ACC	SEN	SPE	PPV	NPV	AUC	Time	Nfeats
GARS	1	1	1	1	1	1	4 min	14
RFE	0.75	0.75	0.75	0.75	0.75	0.94	1 s	5
SBF	1	1	1	1	1	1	15 s	74
rfGA	1	1	1	1	1	1	1 h 33 min	84
svmGA	1	1	1	1	1	1	13 h 2 min	23
LASSO	1	1	1	1	1	1	1 s	14

*ACC* Accuracy, *SEN* Sensitivity, *SPE* Specificity, *PPV* Positive Predictive Value, *NPV* Negative Predictive Value, *AUC* Area Under ROC Curve, *Time* average learning time for each cross-validation fold, *Nfeats* n. of selected features

#### Mid-dimensional dataset in a binary classification problem

A second test consisted of comparing the FS methods on the ‘Binary mid-dimension’ dataset, which was composed of 26 patients affected by Acute Kidney Injury (AKI) and 72 healthy subjects (non-AKI). Using this dataset, we assessed the performance of the 5 algorithms in a hard binary classification problem, where the number of features is pretty high and two groups are not well separated (see Additional file 1: Figure S1, panel B). Here, the ‘learning dataset’ was generated by random sampling of 20 patients with AKI and 20 non-AKI. The remaining 50 non-AKI and 6 AKI samples were used as the ‘independent test set’ for performance evaluation. The GA settings were the same as the previous analysis, except for the number of iteration, set to 150. We considered ‘AKI’ as the positive class.

**Fig. 3:**
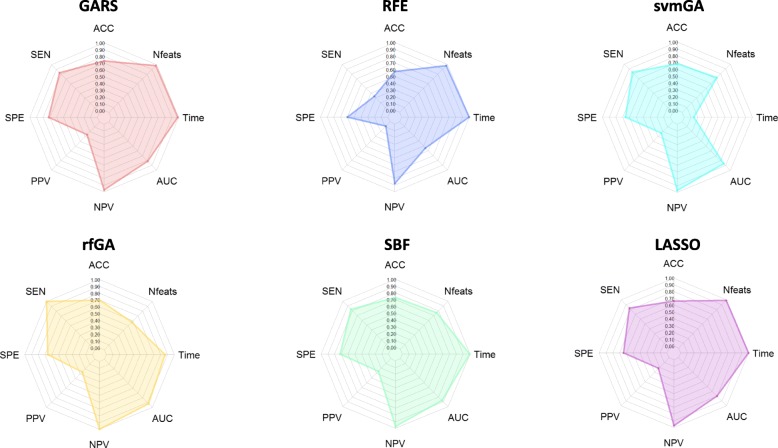
Radar plots that summarize the performance of the different algorithms tested in a ‘binary mid-dimension dataset’. To test the efficacy of each algorithm, we calculated ACC = Accuracy, SEN = Sensitivity, SPE = Specificity, PPV = Positive Predictive Value, NPV = Negative Predictive Value, AUC = Area Under ROC Curve, and Nfeats = n. of selected features on the independent test set. To evaluate the efficiency of each algorithm, we measured the average learning time for each cross-validation fold (Time). To get an overall assessment of the algorithm performance, we calculated the area of the polygon obtained connecting each point of the aforementioned measurements: the wider the area, the better the overall performance. GARS (red chart) covered 62% of the total area, SBF (green chart) 59%, LASSO (purple chart) 58%, rfGA (yellow chart) 55%, RFE (blue chart) 39% and svmGA (light blue chart) 38%

On this dataset, GARS found a feature set that allowed reaching the highest classification accuracy (73%) and the best compromise between sensitivity and specificity with a small number of features (*n* = 7; see Table 2). Conversely, SBF, which showed similar accuracy and performance, identified a minimum feature set of 83 metabolites; and, LASSO, which selected the smallest number of features (*n* = 2; Table 2) but at the expense of a relevant lower accuracy (66%) compared to GARS. In terms of computational learning time, GARS dramatically outperformed the other two GAs: rfGA and svmGA took 2–16 h per fold to complete the analysis, while GARS less than 12 min. The radar chart in Fig. 3 summarizes these results: GARS covered a larger area (62%) than any other algorithm, which ranged from 38 to 59%.

**Table 2:** Performance evaluation, testing FS methods on the ‘binary mid-dimension’ dataset

	ACC	SEN	SPE	PPV	NPV	AUC	Time	Nfeats
GARS	0.73	0.83	0.72	0.26	0.97	0.81	11 min 41 s	7
RFE	0.57	0.33	0.6	0.09	0.88	0.54	2 s	10
SBF	0.73	0.83	0.72	0.26	0.97	0.87	20 s	83
rfGA	0.7	1	0.66	0.26	1	0.92	2 h 33 min	145
svmGA	0.68	0.83	0.66	0.23	0.97	0.86	16 h 53 min	94
LASSO	0.66	0.83	0.64	0.22	0.97	0.80	1 s	2

*ACC* Accuracy, *SEN* Sensitivity, *SPE* Specificity, *PPV* Positive Predictive Value, *NPV* Negative Predictive Value, *AUC* Area Under ROC Curve, *Time* average learning time for each cross-validation fold, *Nfeats* n. of selected features

#### High-dimensional datasets in multi-class classification problems

For the last machine learning analysis, we picked samples belonging to 11 brain regions from a large normal tissue transcriptomics dataset, with a total of 19,162 features. This high-dimensional dataset was used to test the FS algorithms in multi-class classification problems, where the number of features is as high as in common RNA-Seq datasets, and each group is very similar to each other (see Additional file 1: Figure S1, panel C). We constructed five different datasets, composed of an increasing number of tissue samples (from 3 to 11 with 2-step increments), and generated ‘learning datasets’ by random sampling 50 samples per tissue. The remaining samples (*n* = 156–479) were used as ‘independent test sets’ for performance evaluation. The GA settings were the same as the previous analysis, except for the desired chromosomal feature range that was set from 15 to 25.

The performance achieved by GARS were very high in all multi-class analyses, as shown in Table 3: accuracies ranged from 0.86 to 0.92, decreasing linearly (*r* = − 0.96, *p* = 0.009) as the number of classes increased. We observed similar inverse correlations between the number of classes and sensitivity (*r* = − 0.96, *p* = 0.01), specificity (*r* = 0.88, *p* = 0.05), PPV (*r* = − 0.96, *p* = 0.01), NPV (*r* = 0.85, *p* = 0.07), number of features (*r* = 0.88, *p* = 0.05), and learning time expressed on a log2 scale (*r* = 1, *p* < 0.001).

**Table 3:** Performance evaluation, testing GARS on ‘multi-class high-dimension’ datasets

	ACC	SEN	SPE	PPV	NPV	Time	Nfeats
3 classes	0.92	0.89	0.95	0.87	0.94	59 min	15
5 classes	0.91	0.85	0.96	0.84	0.96	1 h 48 min	18
7 classes	0.89	0.82	0.97	0.78	0.97	3 h 43 min	18
9 classes	0.89	0.82	0.97	0.79	0.97	6 h 49 min	24
11 classes	0.86	0.75	0.97	0.72	0.97	11 h 55 min	22

*ACC* Accuracy, *SEN* Sensitivity, *SPE* Specificity, *PPV* Positive Predictive Value, *NPV* Negative Predictive Value, *AUC* Area Under ROC Curve, *Time* average learning time for each cross-validation fold, *Nfeats* n. of selected features

The result for such complex settings clearly revealed the limitations of the other feature selection methods considered. Indeed, we observed that: (i) LASSO, RFE and SBF implementations cannot handle a huge number of variables as produced by RNA-seq experiment (> 15,000); and, (ii) rfGA and svmGA cannot complete the analyses within the time limit of 24 h per fold.

To try and compare GARS with the other tools in a multi-class setting, we reduced the number of features of the five high-dimensional datasets selecting the top 1000 genes with the highest variance over all samples. As summarized in Table 4, again svmGA did not complete the analysis in the maximum time allotted (24 h per fold), whereas rfGA accomplished the task only when the number of classes was equal to 3. Conversely, SBF was able to rapidly select feature sets for any given multi-class analysis, but the number of variables chosen ranged from 28% (3-class problem) to 98% (11-class problem) of the available features. RFE showed the shortest learning time, but in three cases did not perform any feature selection at all (*n* = 999 in 3-, 7-, and 11-class analyses). LASSO showed a classification accuracy of 3–6% higher than GARS; however, the number of features selected by LASSO was from 3 to 7 times higher than those identified by GARS. Overall, although classification accuracy and other metrics were similar whatever the number of classes, the number of selected features was dramatically different. GARS always selected the lowest number of features in all the analyses performed. Notably, when the number of classes was greater than 5, the learning time required by GARS for the feature selection using the full (19,162 genes) or reduced datasets (1000 genes) was not significantly different (*p* = 0.08).

**Table 4:** Performance evaluation, testing FS methods on reduced ‘multi-class high-dimension’ datasets (1000 features)

		ACC	SEN	SPE	PPV	NPV	Time	Nfeats
3	GARS	0.92	0.90	0.95	0.88	0.94	26 min	18
3	RFE	0.94	0.92	0.96	0.91	0.96	3 s	999
3	SBF	0.95	0.94	0.97	0.94	0.97	1 min 58 s	289
3	rfGA	0.93	0.91	0.95	0.91	0.95	19 h 55 min	598
3	svmGA	–	–	–	–	–	–	–
3	LASSO	0.95	0.93	0.96	0.93	0.97	2 s	54
5	GARS	0.93	0.89	0.97	0.89	0.97	1 h 22 min	17
5	RFE	0.93	0.89	0.97	0.88	0.97	6 s	21
5	SBF	0.93	0.89	0.97	0.87	0.97	9 min 38 s	890
5	rfGA	–	–	–	–	–	–	–
5	svmGA	–	–	–	–	–	–	–
5	LASSO	0.96	0.93	0.98	0.93	0.98	2 s	74
7	GARS	0.90	0.84	0.97	0.81	0.97	3 h 6 min	16
7	RFE	0.95	0.91	0.98	0.89	0.98	13 s	999
7	SBF	0.95	0.92	0.99	0.90	0.99	16 min 7 s	959
7	rfGA	–	–	–	–	–	–	–
7	svmGA	–	–	–	–	–	–	–
7	LASSO	0.96	0.93	0.99	0.90	0.99	4 s	105
9	GARS	0.92	0.86	0.98	0.85	0.98	6 h 6 min	22
9	RFE	0.93	0.89	0.98	0.87	0.98	11 s	25
9	SBF	0.95	0.91	0.99	0.89	0.99	22 min 47 s	963
9	rfGA	–	–	–	–	–	–	–
9	svmGA	–	–	–	–	–	–	–
9	LASSO	0.96	0.92	0.99	0.90	0.99	6 s	123
11	GARS	0.93	0.88	0.99	0.86	0.98	10 h 31 min	19
11	RFE	0.94	0.90	0.99	0.88	0.99	17 s	999
11	SBF	0.95	0.91	0.99	0.89	0.99	30 min 44 s	976
11	rfGA	–	–	–	–	–	–	–
11	svmGA	–	–	–	–	–	–	–
11	LASSO	0.96	0.92	0.99	0.90	0.99	9 s	134

*ACC* Accuracy, *SEN* Sensitivity, *SPE* Specificity, *PPV* Positive Predictive Value, *NPV* Negative Predictive Value, *AUC* Area Under ROC Curve, *Time* average learning time for each cross-validation fold, *Nfeats* n. of selected features

### Robustness of GARS

In most comparisons, GARS ensured that the differences between accuracies on a training set and test set (∆) were less than 10%. The only three exceptions are the performance on the mid-dimensional dataset (∆ = 25% [1.8–48.2]) and on the high-dimensional dataset with 11 classes, where ∆ = 12% [10.8–13.2] and ∆ = 10.6% [7.8–13.4], with all features and with the top 1000 most variant features, respectively. Results obtained in all simulations for each FS methods are summarized in Additional file 2.

## Discussion

The ever-increasing development of ground-breaking technologies has changed the way in which data are generated, making measuring and gathering a large number of variables a common practice in science today. Regardless of the field of study, the common but challenging goal for most data analysts is to identify, from this large amount of data, the most informative variables that can accurately describe and address a relevant biological issue, namely, the feature selection. Feature selection is particularly important in the context of classification problems because multivariate statistical models for prediction usually display better performance by using small sets of features than building models with bulks of variables. Unlike other methods of dimensional reduction, the feature selection techniques maintain the original representation of the variables and seek for a subset of them, while concurrently optimizing a primary objective, e.g. prediction performance on future data [24, 25]. Reducing the complexity of high-dimensional data by feature selection has different potential benefits, including (i) limiting overfitting while simplifying models, (ii) improving accuracy and (iii) computational performance, (iv) enabling better sample distinction by clustering, (v) facilitating data visualization and (vi) providing more cost-effective models for future data.

Conversely, the use of an inefficient feature selection strategy can lead to over-fitting or poorly performing classification models. Nonetheless, the feature selection step is underestimated in several applications as common users often prefer to apply fast, easy-to-use techniques instead of methods where multiple parameters have to be set or computational time is high, all at the expense of accuracy and precision. However, the selection of the correct feature selection algorithm and strategy is still a critical challenge [7]. Among feature selection techniques, GA has been proven to be effective as both a dimensional reduction (feature extraction) and feature selection method. Although feature extraction can be very effective in reducing the dimensional space and improving classification performance both in terms of accuracy and speed, it works by transforming the original set of features into new (few) ones. The drawback of this approach is that the extracted features are derived as a combination of the original variables and, therefore, the number of features to be experimentally tested cannot be reduced in practice. This issue is particularly relevant when dealing with Omic data since they are generated by expensive experimental settings. This makes a feature extraction approach less feasible for real-world scenarios where, instead, the use of low-cost measurements of few sensitive variables (e.g. biomarkers) is a challenging target, for example for medical applications [26].

However, class-dependent feature selection by GA has been already shown to perform efficiently and with fast processing on medium-sized datasets (~ 100 features) with similar or even better accuracy compared to well-performing algorithms such as those based on sequential floating forward search [9, 27]. Despite that, the methods based on GA traditionally did not deal with high-dimensional data as produced by the most modern, cutting-edge Omics technologies and, thus, GAs have not been widely used in this context.

By combining a dimension reduction method (i.e. MDS) with a score of similarity (i.e. silhouette index) between well-defined phenotypic sample groups (aka classes), GARS represents an innovative supervised GA implementation that, exploiting the search optimization of population-based algorithms, proves to be an efficient and timely method of selecting informative features on simple (binary) and complex (multi-class) high-dimensional data issues. Actually, other GA implementations have already considered the use of similarity scores to assess the consistency of clustering in an unsupervised setting [28, 29]. The main difference with GARS is that our algorithm is designed to solve a supervised problem where the averaged silhouette index calculation of the MDS result is embedded in the fitness function to estimate how well the class-related phenotypes are grouped together while searching the optimal solution. In addition to being effective, the combination of the MDS and the silhouette index calculations proved to be very fast, thus producing accurate solutions for high-dimensional data sizes as well. On the contrary, the excessive time of execution for other GA implementations (i.e. days) or the inadequacy to handle complex problems (multi-class settings, tens of thousands of variables) preclude their use for real applications.

We demonstrated the GARS efficiency by benchmarking against the most popular feature selection methods, including filter-based, wrapper-based and embedded methods, as well as other GA methods. We showed that GARS enabled the retrieval of feature sets in binary classification problems, which always ensured classification accuracy on independent test sets equal or superior to univariate filter-based, wrapper and embedded methods and other GAs. We also found that the selected features by GARS were robust, as the error rate on the validation test sets was consistently low for GARS and obtained with the lower number of features selected compared to the other methods. Furthermore, for real-time processing, GARS required a computational time that was similar compared to filter-based, wrapper or embedded feature selection methods, or drastically lower, roughly 1% on average, compared to GAs, but always returning a set with the lower number (up to 6 times less) of informative variables.

Remarkably, when dealing with high-dimensional data sets, i.e. presenting around 20,000 features (as in common Omic experiments), GARS was the only method able to complete the analysis on all variables without any pre-filtering step. Specifically, in multi-class classification problems, GARS achieved classification accuracies ranging from 0.86 (11 classes) to 0.92 (3 classes), selecting feature sets with at most 25 variables. Consistently, even if we reduced the number of original variables of the high-dimensional datasets to a smaller one (i.e. 1000 features), enabling comparison with the other feature selection methods, GARS guaranteed similar performance to the other algorithms (accuracies greater than 0.9), but always selecting the smallest set of features.

## Conclusions

While we do not presume to have covered here the full range of options for performing feature selection on high-dimensional data, we believe that our test suggests GARS as a powerful and convenient resource for timely performance of an effective and robust collection of informative features in high-dimensions. Through comparing with other feature selection algorithms, we also showed that GARS is feasible for real-world applications when applying to solve a complex (multi-class) problem. Therefore, GARS could be adopted when standard feature selection approaches do not provide satisfactory results or when there is a huge amount of data to be analyzed.

## Methods

### Dataset collection and pre-processing

To test and compare the performance of the different feature selection algorithms, we collected and pre-processed three publicly available -omics datasets:

- **‘Binary low-dimension’ dataset**. To generate this dataset, we filtered and normalized the data produced by [20], consisting of 58 samples (29 with cervical tumor vs. 29 without tumor) in which the expression of 714 miRNAs was assessed by RNA-Seq technology. Specifically, we discarded features (miRNAs) with less than 10 counts in more than 50% of the samples; subsequently, the variance stabilizing transformation was applied.
- **‘Binary mid-dimension’ dataset.** We derived this dataset from the NMR spectrometry characterization, conducted by [21], of the urine metabolomic profiles in 72 healthy subjects and 34 patients affected by AKI, divided into three classes based on the Acute Kidney Injury Network (AKIN) criteria. The number of metabolic features is 701 and we used the original data normalized by quantile normalization. To accomplish the binary classification task, we selected all the healthy donors and the 26 patients with stage-1 AKI.
- **‘Multi-Class high-dimension’ datasets.** These datasets were yielded exploiting the Genotype-Tissue Expression Project (GTEx) that collects the transcriptome profiles (56,318 transcripts) of 53 tissues gathered from more than 700 donors [22, 23]. We selected samples from 11 brain regions: amygdala (*n* = 72), anterior cingulate cortex (*n* = 84), caudate (*n* = 117), cortex (*n* = 114), frontal cortex (*n* = 108), hippocampus (*n* = 94), hypothalamus (*n* = 96), nucleus accumbens (*n* = 113), putamen (*n* = 97), spinal cord (*n* = 71), and substantia nigra (*n* = 63). We applied the same filtering and normalization steps, adopted for the ‘binary-low dimension’ dataset.

### Comparison and evaluation metrics

**Fig. 4:**
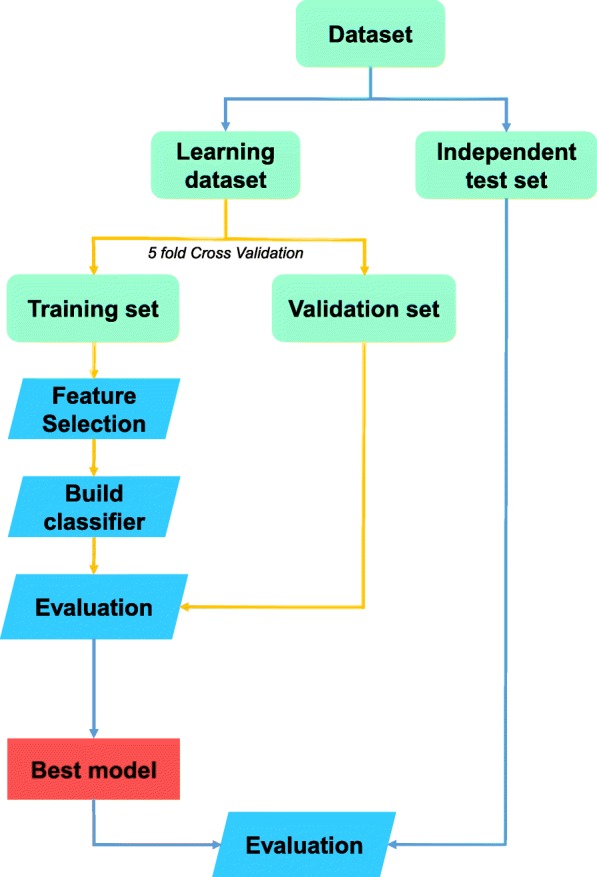
Flowchart of the Machine Learning process used to assess the performance of each algorithm tested. Each dataset is initially split into two subsets: the ‘Learning dataset’ and the ‘Independent test set’. Subsequently, the former undergoes a 5-fold cross validation strategy, where Training sets are used to select informative features (‘Feature Selection’) and Validation sets to test the classifier performance (‘Evaluation’). Finally, the Best Model is selected and, then, assessed on the Independent test set (‘Evaluation’): the last evaluation step is used to compare the performance of each feature selection method

To evaluate the goodness of the FS algorithms, we implemented a supervised machine learning analysis, depicted in Fig. 4. First, we split each dataset into two parts: a balanced set, called “learning dataset” and an “independent test set”. Then, we applied a 5-fold cross-validation strategy to the learning dataset: this was repeatedly subdivided into training sets, used to select informative features and subsequently build a random forest classifier [30], and in validation sets, used to test the classifier performance. Extending the concept of a decision tree, this classifier belongs to the class of ensemble strategy. First, several decision trees are built independently, sampling a bunch of features in a random way. Then, the predictions of each tree are taken into account to perform the random forest classification, weighting each tree by a voting approach. This implementation ensures high accuracy and low over-fitting. For each fold, the number of selected features, the average computational time during the learning steps (Learning Time), accuracy, specificity, sensitivity (i.e.*,* recall), PPV and NPV (i.e.*,* precision) were calculated for each validation set. For binary comparisons, the area under the ROC curve (AUC) was also computed [31]. Finally, based on the highest AUC (binary comparisons) or the highest accuracy (multi-class comparisons) and the lowest number of features selected, we chose the best model: this was successively tested on the independent test set [32], measuring accuracy, specificity, sensitivity, PPV, NPV, and AUC when appropriate (see Tables 1, 2, 3, and 4). We excluded from the comparisons those feature selection algorithms that (*a*) took more than 24 h per fold to produce the results and/or (*b*) stopped the execution because of the high number of features to work with.

To get an overall view of the results of the binary classification analysis, we drew radar-plots. These graphs are composed of equiangular radii on a circumference, where each segment represents a specific measurement.

In order to set the same range used for the machine learning evaluation metrics, values corresponding to the number of features and the computational time were scaled between 0 and 1. We calculated the area covered *A*_*cov*_ by the polygon obtained connecting the endpoints of each segment, by the formula:

3 $$ {A}_{cov}=\left(\frac{1}{2}\times {r}_n\times {r}_1\times \sin \left(\gamma \right)\right)+{\sum}_{1=1}^{n-1}\left(\frac{1}{2}\times {r}_i\times {r}_{i+1}\times \sin \left(\gamma \right)\right) $$

where *i* represents the magnitude of the *i* measurement, *γ* is the angle between two consecutive radii, and *n* is the number of measurements. Finally, the performance is evaluated by the ratio between *A*_*cov*_ and the total area available (*A*_*max*_):

4 $$ {A}_{\%}=\frac{A_{cov}}{A_{max}}\times 100 $$

where:

5 $$ {A}_{max}=\frac{n}{2}\times \sin \left(\gamma \right) $$

### Robustness analysis

In machine learning, the robustness is the property of a classifier or a classification analysis to ensure similar performances on both training and test sets. The lower this difference in performance, the more robust a classification. Therefore, we evaluated the robustness of each feature selection tool, retrieving their performances on training and validation sets during the 5-fold cross-validation. In particular, we assessed the robustness by computing the average difference in accuracy (∆) and the 95% confidence intervals over the five iterations.

### Tools for data handling and assessments

GARS was entirely created in R v.3.5.1 environment [33]. The filtering and normalization steps were performed using the ‘DaMiRseq’ package [34]. Performances were assessed by the dedicated functions, implemented in the ‘caret’ package [12]. LASSO, rfGA, svmGA, RFE, and SBF were performed by exploiting the dedicated functions, implemented in the ‘caret’ package [12]. Radar-plots were drawn using the ‘fmsb’ R package.

### Hardware resources

All the analyses were run on R, installed in Windows 10 on a Workstation that has 64 GB of RAM and an Intel® Xeon® CPU ES-2623 v4 @ 2.60 GHz processor.

## Availability and requirements

Project name: GARS.

Project home page: https://www.bioconductor.org/packages/GARS/

Operating system(s): platform-independent.

Programming language: R.

Other requirements: none.

License: GLP (> = 2).

Any restrictions to use by non-academics: No restrictions

## Supplementary information

**Additional file 1. **MDS plots. A MDS plot for each dataset is provided. **Figure S1.** Multi-Dimensional Scaling plots. Multi-Dimensional Scaling analyses were applied to display normalized datasets in a two-dimensional space and to highlight the degree of separation between groups. In the ‘binary low-dimension’ dataset (panel A), tumor samples (T, blue triangles) and non-tumor (NT, red circles) are well separated. Conversely, samples composing the ‘binary mid-dimension’ dataset (panel B) are quite mixed (AKI, red circles, and non-AKI, blue triangles). Concerning the ‘multi-class high-dimension’ dataset (panel C), samples from the 11 brain tissues are substantially overlapped, with the exception of those from the spinal cord.

**Additional file 2.** Results of the robustness analysis. In this file, we summarized the results of the robustness analysis, performed for each dataset. A table is provided for each analysis.

## Data Availability

GARS is a Bioconductor package, consisting of a set of functions that allows building a user-tailored GA to find informative variables. GARS was developed in the R environment (R ≥ 3.5) and was released under GPL (≥ 2) License. The package runs on Windows, Linux and Macintosh operating systems and is freely available to non-commercial users at https://github.com/BioinfoMonzino/GARS and at the Bioconductor open-source, open-development software project repository (https://bioconductor.org/packages/GARS/). In compliance with Bioconductor standards, the authors ensure stable package maintenance through software and documentation updates. The code implemented to perform the analysis is deposited at https://github.com/BioinfoMonzino/GARS_paper_Code The datasets supporting the conclusions of this article are available in the following sources: Witten et al. [20], https://static-content.springer.com/esm/art%3A10.1186%2F1741-7007-8-58/MediaObjects/12915_2010_354_MOESM2_ESM.xls; MetaboLights [35], ftp://ftp.ebi.ac.uk/pub/databases/metabolights/studies/public/MTBLS24/AKI_quantile_norm_16_10_2012.csv ;and, GTEx [22, 23], https://storage.googleapis.com/gtex_analysis_v6/rna_seq_data/GTEx_Analysis_v6_RNA-seq_RNA-SeQCv1.1.8_gene_reads.gct.gz.

## Abbreviations

ACC: Accuracy
AKI: Acute Kidney Injury
AKIN: Acute Kidney Injury Network
aSI: average Silhouette Index
AUC: Area Under ROC Curve
FS: Feature Selection
GA: Genetic Algorithm
GARS: Genetic Algorithm for the identification of a Robust Subset of features
GTEx: Genotype-Tissue Expression portal
LASSO: Least Absolute Shrinkage and Selection Operator
MDS: Multi-Dimensional Scaling
miRNA: micro RNA
miRNA-Seq: micro RNA Sequencing
Nfeats: Number of selected features.
NMR: Nuclear Magnetic Resonance
non-AKI: non Acute Kidney Injury
NPV: Negative Predictive Value
NT: non tumors
PPV: Positive Predictive Value
RFE: Recursive 385 Feature Elimination
rfGA: ‘Random Forest’-based Genetic Algorithm
RNA-Seq: RNA Sequencing
ROC: Receiver Operating Characteristic
SBF: Selection By Filtering
SEN: Sensitivity
SPE: Specificity
svmGA: ‘Support Vector Machine’-based Genetic Algorithm
T: tumors

## References

[1] Kourou K, Exarchos TP, Exarchos KP, Karamouzis MV, Fotiadis DI (2015). Machine learning applications in cancer prognosis and prediction. Comput Struct Biotechnol J. Elsevier.

[2] Antman EM, Loscalzo J (2016). Precision medicine in cardiology. Nat Rev Cardiol. Nat Publ Group.

[3] Wang L, Chu F, Xie W (2007). Accurate cancer classification using expressions of very few genes. IEEE/ACM Trans Comput Biol Bioinforma.

[4] Bolón-Canedo V, Sánchez-Maroño N (2016). Alonso-Betanzos A.

[5] Saeys Y, Inza I, Larrañaga P. A review of feature selection techniques in bioinformatics. Bioinformatics. Oxford University Press; 2007;23:2507–2517. 10.1093/bioinformatics/btm344 17720704

[6] Hira Zena M., Gillies Duncan F. (2015). A Review of Feature Selection and Feature Extraction Methods Applied on Microarray Data. Advances in Bioinformatics.

[7] Perez-Riverol Yasset, Kuhn Max, Vizcaíno Juan Antonio, Hitz Marc-Phillip, Audain Enrique (2017). Accurate and fast feature selection workflow for high-dimensional omics data. PLOS ONE.

[8] Wang L, Wang Y, Chang Q (2016). Feature selection methods for big data bioinformatics: a survey from the search perspective. Methods..

[9] Oh IS, Lee JS, Moon BR (2004). Hybrid genetic algorithms for feature selection. IEEE Trans Pattern Anal Mach Intell.

[10] Zawbaa HM, Emary E, Grosan C, Snasel V (2018). Large-dimensionality small-instance set feature selection: a hybrid bio-inspired heuristic approach. Swarm Evol Comput Elsevier.

[11] Mohamad MS, Deris S, Illias RM (2005). A hybrid of genetic algorithm and support vector machine for features selection and classification of gene expression microarray. Int J Comput Intell Appl World Scientific.

[12] Kuhn M (2008). Others. Building predictive models in R using the caret package. J Stat Softw.

[13] Rousseeuw PJ (1987). Silhouettes: a graphical aid to the interpretation and validation of cluster analysis. J Comput Appl Math Elsevier.

[14] Holland JH. Adaptation in natural and artificial systems: an introductory analysis. Adapt. Nat. Artif. Syst. An Introd. Anal. with Appl. to Biol. Control. Artif. Intell. 1975.

[15] Goldberg D. Genetic algorithms in search, optimization, and machine learning. Choice Rev Online. 1989.

[16] Guyon I, Weston J, Barnhill S, Vapnik V. Gene selection for cancer classification using support vector machines. Mach Learn. 2002;

[17] Tibshirani R. Regression shrinkage and selection via the Lasso. J R Stat Soc Ser B. 1996.

[18] Khazaee A, Ebrahimzadeh A. Classification of electrocardiogram signals with support vector machines and genetic algorithms using power spectral features. Biomed Signal Process Control. 2010;

[19] Scrucca L. GA: a package for genetic algorithms in R. J Stat Softw 2013;

[20] Witten Daniela, Tibshirani Robert, Gu Sam, Fire Andrew, Lui Weng-Onn (2010). Ultra-high throughput sequencing-based small RNA discovery and discrete statistical biomarker analysis in a collection of cervical tumours and matched controls. BMC Biology.

[21] Zacharias HU, Schley G, Hochrein J, Klein MS, Köberle C, Eckardt K-U (2013). Analysis of human urine reveals metabolic changes related to the development of acute kidney injury following cardiac surgery. Metabol Springer.

[22] Lonsdale J, Thomas J, Salvatore M, Phillips R, Lo E, Shad S (2013). The genotype-tissue expression (GTEx) project. Nat Genet. Nat Publ Group.

[23] Consortium Gte. The Genotype-Tissue Expression (GTEx) pilot analysis: multitissue gene regulation in humans. Science (80- ). American Association for the Advancement of Science; 2015;348:648–660. 10.1126/science.1262110 PMC4547484 25954001

[24] Iguyon I, Elisseeff A. An introduction to variable and feature selection. J Mach Learn Res 2003.

[25] Guyon I, Aliferis C, Elissee, A. Causal Feature Selection. 2007.

[26] Raymer ML, Punch WF, Goodman ED, Kuhn LA, Jain AK. Dimensionality reduction using genetic algorithms. IEEE Trans Evol Comput. 2000;

[27] Fu X, Wang L. A GA-based novel RBF classifier with class-dependent features. Proc 2002 Congr Evol Comput CEC 2002. 2002.

[28] Lletí R, Ortiz MC, Sarabia LA, Sánchez MS. Selecting variables for k-means cluster analysis by using a genetic algorithm that optimises the silhouettes. Anal Chim Acta. 2004;

[29] Pan H, Zhu J, Han D (2003). Genetic algorithms applied to multi-class clustering for gene expression data. Genomics Proteomics Bioinformatics.

[30] Breiman L (2001). Random forests. Mach Learn Springer.

[31] Sokolova M, Lapalme G (2009). A systematic analysis of performance measures for classification tasks. Inf Process Manag.

[32] Raschka S. Model evaluation , model selection , and algorithm selection in machine learning Performance Estimation : Generalization Performance Vs . Model Selection arXiv 2018;

[33] R Core Team. R: A Language and Environment for Statistical Computing [Internet]. Vienna, Austria; 2018. Available from: https://www.r-project.org

[34] Chiesa M, Colombo GI, Piacentini L. DaMiRseq -an R/bioconductor package for data mining of RNA-Seq data: normalization, feature selection and classification. Bioinformatics. 2018:34. 10.1093/bioinformatics/btx795 29236969

[35] Haug Kenneth, Salek Reza M., Conesa Pablo, Hastings Janna, de Matos Paula, Rijnbeek Mark, Mahendraker Tejasvi, Williams Mark, Neumann Steffen, Rocca-Serra Philippe, Maguire Eamonn, González-Beltrán Alejandra, Sansone Susanna-Assunta, Griffin Julian L., Steinbeck Christoph (2012). MetaboLights—an open-access general-purpose repository for metabolomics studies and associated meta-data. Nucleic Acids Research.

