# Supplementary figures and images for: GARS: Genetic Algorithm for the identification of a Robust Subset of features in high-dimensional datasets
Source: BMC Bioinformatics. 2020 Feb 11;21:54. doi: 10.1186/s12859-020-3400-6 (PMC7014945; doi:10.1186/s12859-020-3400-6)

**A**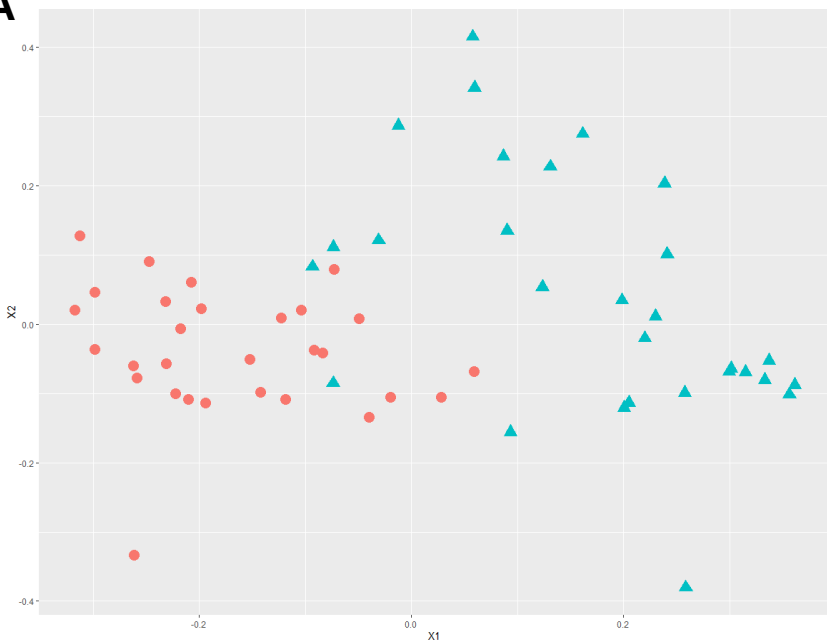**B**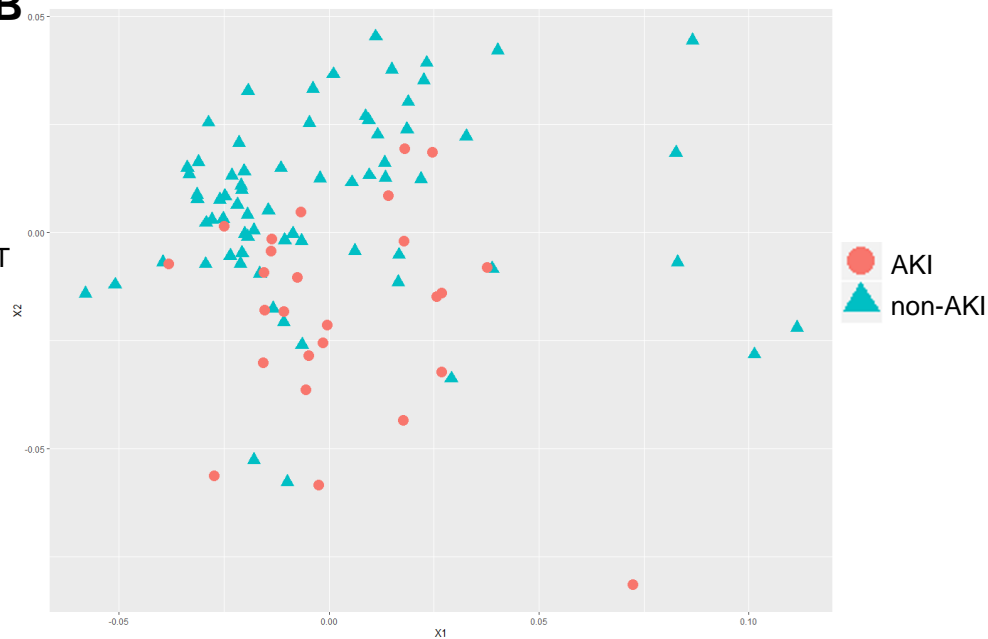**C**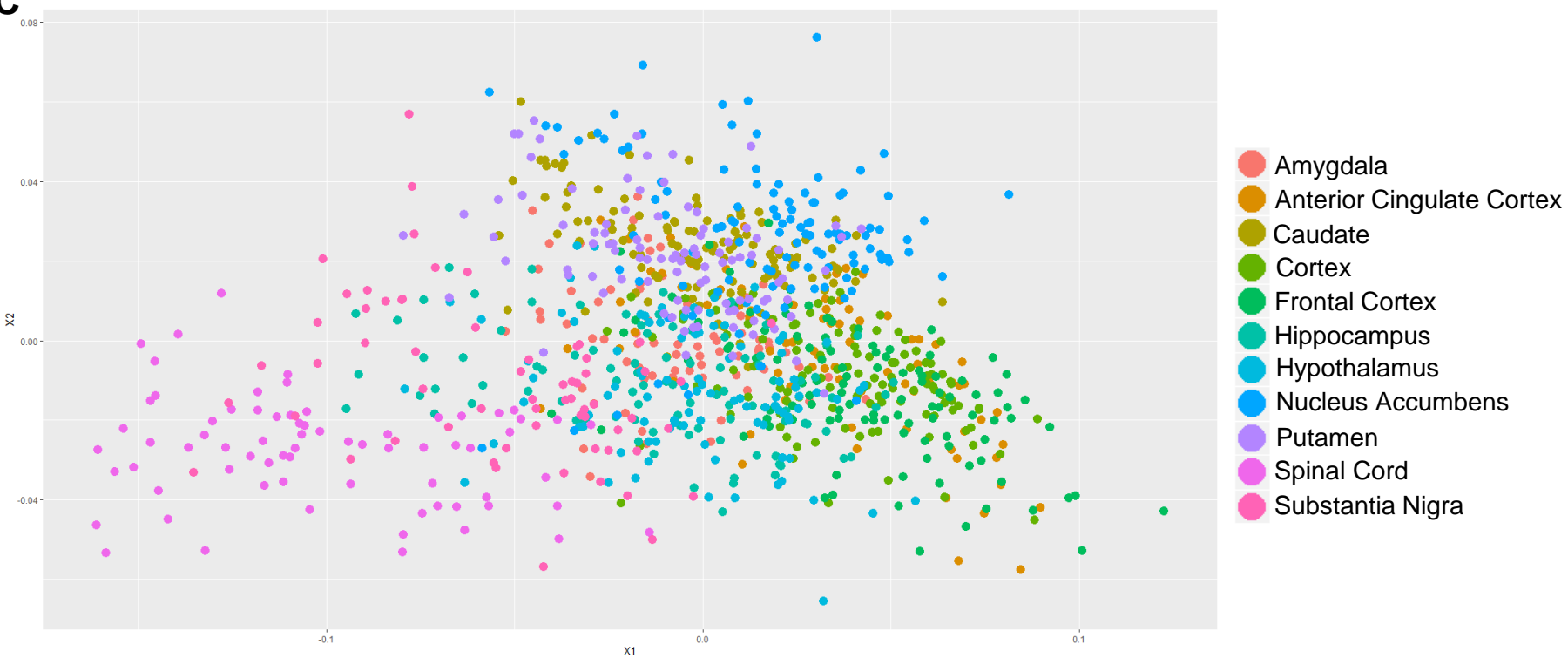

Supplement: Supplementary file 1 — Additional file 1. MDS plots. A MDS plot for each dataset is provided. Figure S1. Multi-Dimensional Scaling plots. Multi-Dimensional Scaling analyses were applied to display normalized datasets in a two-dimensional space and to highlight the degree of separation between groups. In the ‘binary low-dimension’ dataset (panel A), tumor samples (T, blue triangles) and non-tumor (NT, red circles) are well separated. Conversely, samples composing the ‘binary mid-dimension’ dataset (panel B) are quite mixed (AKI, red circles, and non-AKI, blue triangles). Concerning the ‘multi-class high-dimension’ dataset (panel C), samples from the 11 brain tissues are substantially overlapped, with the exception of those from the spinal cord. [file 12859_2020_3400_MOESM1_ESM.pdf]
